# ﻿Description of three new species of the spider genus *Pseudopoda* Jäger, 2000 (Araneae, Sparassidae) from China, Laos and Thailand, and the female of *P.kavanaughi* Zhang, Jäger & Liu, 2023

**DOI:** 10.3897/zookeys.1202.116007

**Published:** 2024-05-27

**Authors:** Yanrong Wu, Rui Zhong, Yang Zhu, Peter Jäger, Jie Liu, He Zhang

**Affiliations:** 1 Hubei Key Laboratory of Regional Development and Environmental Response, Faculty of Resources and Environmental Science, Hubei University, Wuhan 430062, Hubei, China; 2 The State Key Laboratory of Biocatalysis and Enzyme Engineering of China, College of Life Science, Hubei University, Wuhan 430062, Hubei, China; 3 Arachnology, Senckenberg Research Institute, Mertonstraße 17–21, 60325 Frankfurt am Main, Germany; 4 School of Nuclear Technology and Chemistry & Biology, Hubei University of Science and Technology, Xianning 437100, Hubei, China

**Keywords:** Heteropodinae, high diversity, Huntsman spiders, taxonomy

## Abstract

With 252 species, *Pseudopoda* Jäger, 2000, is the largest genus in the family Sparassidae and is widely distributed in South (49 species in Bhutan, India, Nepal and Pakistan), East (158 species in China and Japan) and Southeast Asia (51 species in Indonesia, Laos, Myanmar, Thailand and Vietnam). Few species have been found in more than one region. In this paper, three new species of *Pseudopoda* are described from East and Southeast Asia. Among them, one from China: *P.fengtongzhaiensis* Jäger & Liu, **sp. nov.** (♀); one from Laos: *P.baimai* Jäger & Liu, **sp. nov.** (♀); and one from Thailand: *P.inthanonensis* Jäger & Liu, **sp. nov.** (♀). Additionally, the female of *P.kavanaughi* Zhang, Jäger & Liu, 2023 is described for the first time. Photos of the habitus and genitalia, as well as a distribution map of all four species, are provided.

## ﻿Introduction

The spider genus *Pseudopoda*, established by Jäger (2000) with *Pseudopodaprompta* (O. Pickard-Cambridge, 1885) as the type species, has a broad distribution across South, East and Southeast Asia. Up to now, 252 species have been reported (World Spider Catalog 2024), with their habitats spanning elevations from 17 m (e.g., *P.spirembolus* Jäger & Ono, 2002) (Jäger and Ono 2002) to 3847 m (e.g., *P.nyingchiensis* Zhao & Li, 2018) (Jiang et al. 2018) above sea level. These spiders inhabit a variety of environments, including foliage, leaf litter, rock crevices, caves, grasslands, under tree bark and stones or synanthropic environments. Most species of this genus typically have a limited distribution range, but at the same time, the genus has a high local diversity (Jiang et al. 2018). Researchers speculate that the actual number of *Pseudopoda* species may far exceed those formally described, hinting at the genus’s rich yet partially uncovered biodiversity (Cao et al. 2016; Jiang et al. 2018; Zhang et al. 2023).

To date, nine species groups have been established, primarily based on morphological and molecular characteristics (*daliensis*-group, *interposita*-group, and *signata*-group) or solely on morphological features (*diversipunctata*-group, *latembola*-group, *martensi*-group, *parvipunctata*-group, *prompta*-group, and *schwendingeri*-group) (Jäger 2001; Zhang et al. 2017, 2019; Li et al. 2019). Among these, the *schwendingeri*-group was based only on male characteristics.

One of the notable challenges in studying *Pseudopoda* spiders is the difficulty in collecting mature pairs from the field, leading to many species being described only based on one sex. Currently, more than half of the known species (130) have been documented in this manner. In the course of our research, we have identified three new species from Asia: *P.baimai* Jäger & Liu, sp. nov., *P.fengtongzhaiensis* Jäger & Liu, sp. nov., and *P.inthanonensis* Jäger & Liu, sp. nov. However, only female specimens of these species have been reported. Additionally, during examination of material collected from Kongdang Village, Yunnan Province, China, we found the female of *P.kavanaughi* Zhang, Jäger & Liu, 2023 (Zhang et al. 2023), enabling us to describe the female for the first time in this paper. This study enriches our knowledge of the diversity of the genus *Pseudopoda*.

## ﻿Material and methods

All specimens were kept in 75% ethanol and examined with an Olympus SZX16 stereomicroscope; details were further investigated with an Olympus BX51 compound microscope. Colouration is described in all species from specimens in ethanol. Copulatory organs were examined and illustrated after dissection from the spider bodies; epigynes were cleared with Proteinase K. Habitus photos were obtained with a Leica DMC4500 digital camera attached to a Leica M205 C digital microscope. Coordinates are given in square brackets when retrieved secondarily from Google Earth. A molecular sample ID and collection acronym is given in brackets.

Leg measurements are shown as total length (femur, patella, tibia, metatarsus, tarsus). The number of spines is listed for each segment in the following order: prolateral, dorsal, retrolateral, ventral (in femora and patellae ventral spines are absent and fourth digit is omitted in the spination formula). The terminology used in text and figure legends follows Jäger (2007). All measurements are given in millimetres.

All specimens treated in the present paper were compared with individuals of described species within a certain distribution range to avoid describing synonyms (e.g., *P.nankunensis* Zhang, Jäger & Liu, 2023 in Zhang et al. 2023).

Abbreviations used in text and figures: **AB**, anterior bands; **ALE**, anterior lateral eyes; **AME**, anterior median eyes; **CH**, clypeus height; **CO**, copulatory opening; **DS**, dorsal shield of prosoma; **EF**, epigynal field; **FD**, fertilization duct; **Fe**, femur; **FW**, first winding; **IDS**, internal duct system; **LL**, lateral lobes; **Mt**, metatarsus; **OS**, Opisthosoma; **Pa**, patella; **PLE**, posterior lateral eyes; **PME**, posterior median eyes; **Pp**, palp; **S**, spermathecae; **Ti**, tibia; **I**, **II**, **III**, **IV**, legs I to IV.

Collections (with curators): **CAS**, California Academy of Science, San Francisco, California, USA (L. Esposito); **CBEE**, Centre for Behavioural Ecology and Evolution, College of Life Sciences, Hubei University, Wuhan, China (J. Liu); **MHNG**, Muséum d’Histoire Naturelle, Geneve, Switzerland (P.J. Schwendinger); **SMF**, Senckenberg Research Institute, Frankfurt, Germany (P. Jäger).

## ﻿Results

### ﻿Taxonomy

**Family Sparassidae Bertkau, 1872**

**Subfamily Heteropodinae Thorell, 1873**

#### ﻿Genus Pseudopoda

Taxon classificationAnimaliaAraneaeSparassidae

Jäger, 2000

07B273F3-CEF6-5558-9542-1F62FD6EF97D

##### Type species.

*Sarotespromptus* O. Pickard-Cambridge, 1885.

##### Diagnosis.

Small to large Heteropodinae. Species can be diagnosed by: 1) conductor membranous (some species reduced or entirely absent); 2) embolus broadened and flattened (at least in its proximal part); 3) retrolateral tibial apophysis arising proximally or mesially from the tibia; 4) lateral lobes of epigyne distinctly extend beyond the epigastric furrow, covering the median septum in most species; 5) first winding membranous, with bent margins in most species; and 6) first winding or first winding and lateral lobes covering the internal duct system in dorsal view (modified from Jäger 2000, Jiang et al. 2018 and Zhang et al. 2023).

#### ﻿ Pseudopoda baimai

Taxon classificationAnimaliaAraneaeSparassidae

Jäger & Liu sp. nov.

5393AAB5-6492-5C17-82B3-093936B21BAE

https://zoobank.org/A0DC4815-BE5C-48D8-B349-524C54B51117

Figs 1 , 2 , 9

##### Type material.

***Holotype*** female: Laos, Vientiane Province: Vang Vieng, W of Nam Song, Tham Nam Or Kjem, in cave, 18°55'46.86"N, 102°20'56.82"E, 324 m, 28 July 2016, by hand, by day. P. Jäger leg. (SMF, LAO0008). ***Paratype***: 1 female, with same data as for holotype (SMF, LAO0008).

##### Etymology.

The specific name is derived from the Laos word baimai (ໃບໄມ້), meaning foliage and referring to the fact that the holotype female was collected on foliage of a secondary forest; noun in apposition.

##### Diagnosis.

The female of *P.baimai* Jäger & Liu, sp. nov. resembles *P.gongschana* Jäger & Vedel, 2007 (Jäger and Vedel 2007) by: 1) IDS visible through cuticle in ventral view as large circular patches in centre of LL; and 2) posterior margins of LL with median indentation. It can be recognised by: 1) S largely covered by LL in dorsal view; and 2) anterior margins of LL straight, almost “V”-shaped in ventral view, their lateral ends with a bend, laterad (covered by FW, anterior margins distinctly convex, their lateral ends latero-posteriad in *P.gongschana*).

**Female** (LAO0008): ***Measurements***: Medium sized. Body length 11.3, DS length 4.6, width 4.0, OS length 6.7, width 3.8. ***Eyes***: AME 0.12, ALE 0.26, PME 0.22, PLE 0.24, AME–AME 0.16, AME–ALE 0.08, PME–PME 0.28, PME–PLE 0.35, AME–PME 0.33, ALE–PLE 0.30, CHAME 0.40, CHALE 0.34. ***Spination***: Pp 131, 101, 2121, 1014; Fe I–III 323, IV 331; Pa I–III 001, IV 000; Ti I–II 2226, III–IV 2026; Mt I–II 2024, III 3024, IV 3036. ***Measurements of palps and legs***: Pp 7.1 (2.0, 1.0, 1.6, –, 2.5); I 25.9 (7.7, 2.1, 7.4, 7.0, 1.7); II 28.7 (8.2, 2.5, 8.4, 7.4, 2.2); III 22.2 (6.3, 1.9, 6.8, 5.9, 1.3); IV 24.4 (7.9, 1.2, 7.0, 7.3, 2.0). Leg formula: II-I-IV-III. Promargin of chelicerae with three teeth, retromargin with four teeth, cheliceral furrow with c. 33 denticles.

**Figure 1.**
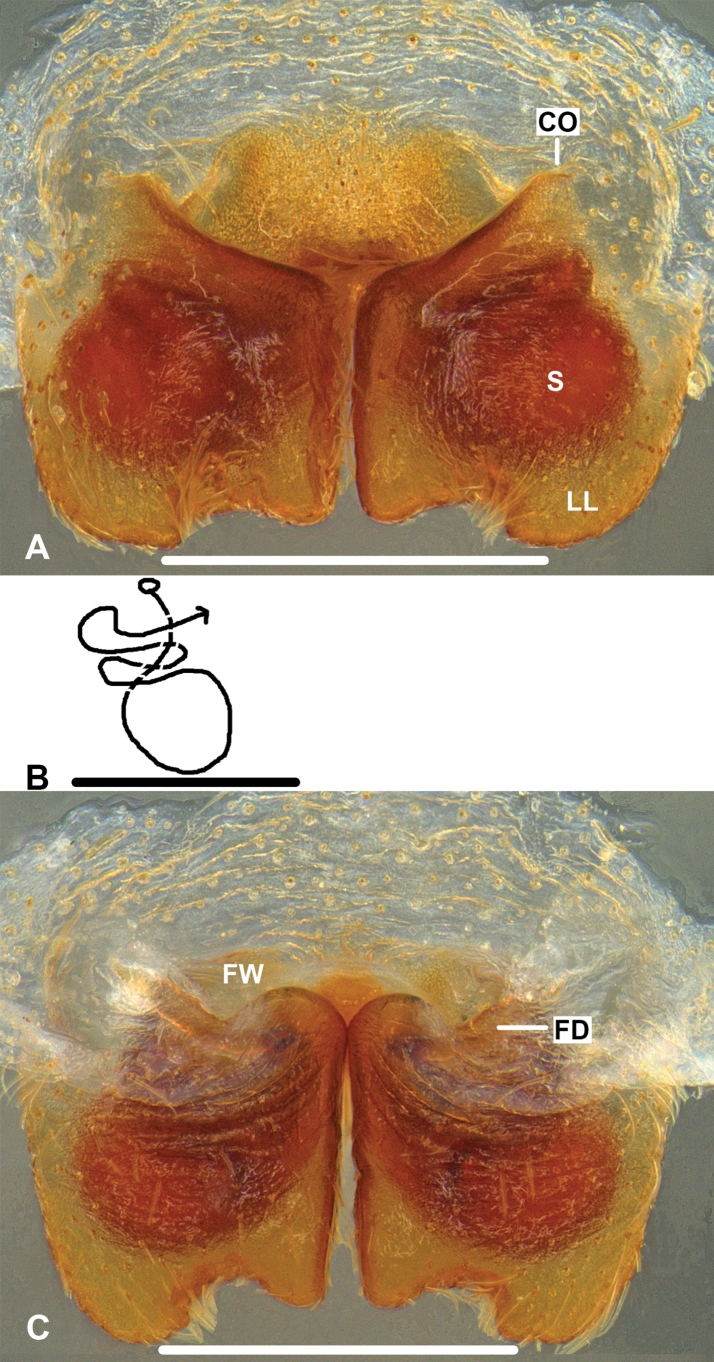
*Pseudopodabaimai* Jäger & Liu, sp. nov., holotype, female **A** epigyne, ventral **B** schematic course of IDS, dorsal **C** vulva, dorsal. Scale bars: 0.5 mm.

***Epigyne*** (Fig. 1A–C): As in diagnosis. EF wider than long, without AB. Posterior margins of LL irregularly rounded. S oval-shaped, occupying most part of LL in ventral view. Most parts of FW covered by LL in dorsal view.

**Figure 2.**
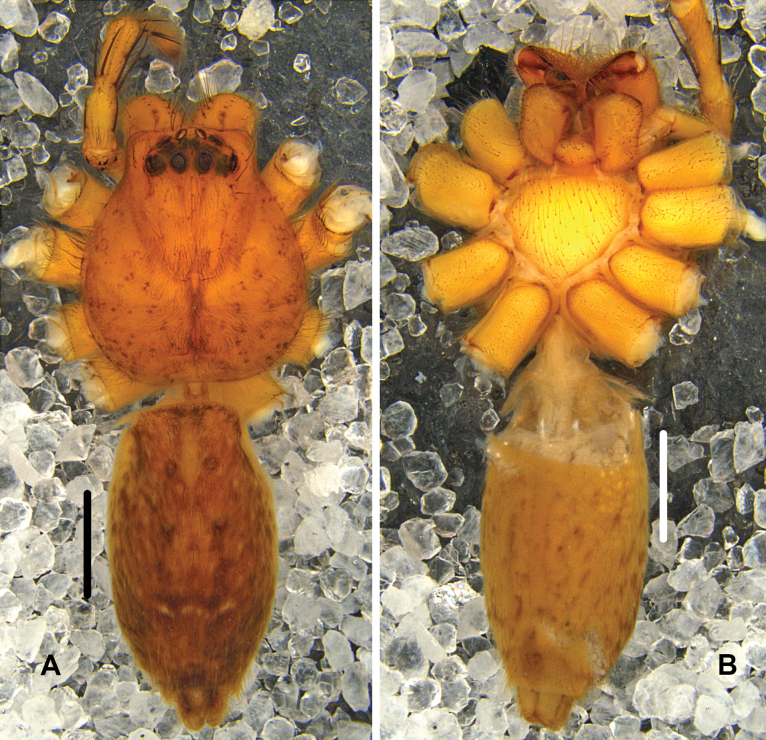
*Pseudopodabaimai* Jäger & Liu, sp. nov., female habitus (**A** dorsal **B** ventral). Scale bars: 2 mm.

***Colouration*** (Fig. 2A, B): DS yellow with dark spots. Fovea distinct. Margin with dark marks. OS dorsally yellowish brown with two light yellow regions at posterior part, ventrally light yellow, with several brown marks, irregularly arranged.

**Male**: Unknown.

##### Variation.

Female (*N* = 1): body length 12.7, DS length 5.3, OS length 7.4.

##### Remarks.

This species might potentially be conspecific with *P.caudata* Zhang, Jäger & Liu, 2023 (Zhang et al. 2023), given that the two habitats are only about 100 kilometres apart. However, there are subtle differences in coloration and patterns on the dorsal side of the females compared to the males of *P.caudata*, indicating a possible distinction between the two species. Further research and future findings are needed to conclusively resolve this ambiguity.

##### Distribution.

Laos (Vientiane Province) (Fig. 9).

#### ﻿ Pseudopoda fengtongzhaiensis

Taxon classificationAnimaliaAraneaeSparassidae

Jäger & Liu sp. nov.

CD9AACD9-1130-50DD-8187-351A699B463F

https://zoobank.org/FE214929-DE51-4FDC-9314-6150FAE50863

Figs 3 , 4 , 9

##### Type material.

***Holotype*** female: China, Sichuan Province: Ya’an City, Baoxing County, Fengtongzhai National Nature Reserve, 30°34′17″N, 102°52′58″E, 1604 m, 5 May 2016, Y. Zhong leg. (CBEE, LJ2215). ***Paratypes***: 3 females, with same data as for holotype (CBEE, LJ2216–LJ2218).

##### Etymology.

The specific name is derived from the type locality, the Fengtongzhai National Nature Reserve; adjective.

##### Diagnosis.

The female of *P.fengtongzhaiensis* Jäger & Liu, sp. nov. is similar to that of *P.emei* Zhang, Zhang & Zhang, 2013 (Zhang et al. 2013) by: 1) LL large, and the length of the lateral margin of LL almost equal to that of median margin in ventral view; and 2) FW well developed. It can be distinguished by: 1) S with a distinct turning in ventral view; and 2) posterior margins of LL rounded and smooth in ventral view (S simple and close to anterior margins of LL, posterior margins of LL with distinct posterior incisions in *P.emei*).

**Female** (LJ2215): ***Measurements***: Medium sized. Body length 15.8, DS length 7.8, width 6.5, OS length 8.0, width 4.9. Eyes: AME 0.22, ALE 0.37, PME 0.24, PLE 0.31, AME–AME 0.13, AME–ALE 0.07, PME–PME 0.18, PME–PLE 0.44, AME–PME 0.27, ALE–PLE 0.23, CHAME 0.38, CHALE 0.25. ***Spination***: Pp 131, 101, 2121, 1014; Fe I–II 323, III–IV 322; Pa I–III 101, IV 100; Ti I 1016, II–IV 2026; Mt I–II 2024, III 3025, IV 3036. ***Measurement of palps and legs***: Pp 8.8 (2.5, 1.2, 1.9, –, 3.2); I 25.3 (7.6, 2.3, 7.4, 6.2, 1.8), II 27.9 (8.0, 2.9, 8.3, 6.6, 2.1), III 20.4 (6.8, 1.6, 5.6, 4.9, 1.5), IV 24.7 (7.8, 1.7, 6.8, 6.4, 2.0). Leg formula: II-I-IV-III. Promargin of chelicerae as in *P.baimai* Jäger & Liu, sp. nov., cheliceral furrow with c. 24 denticles.

**Figure 3.**
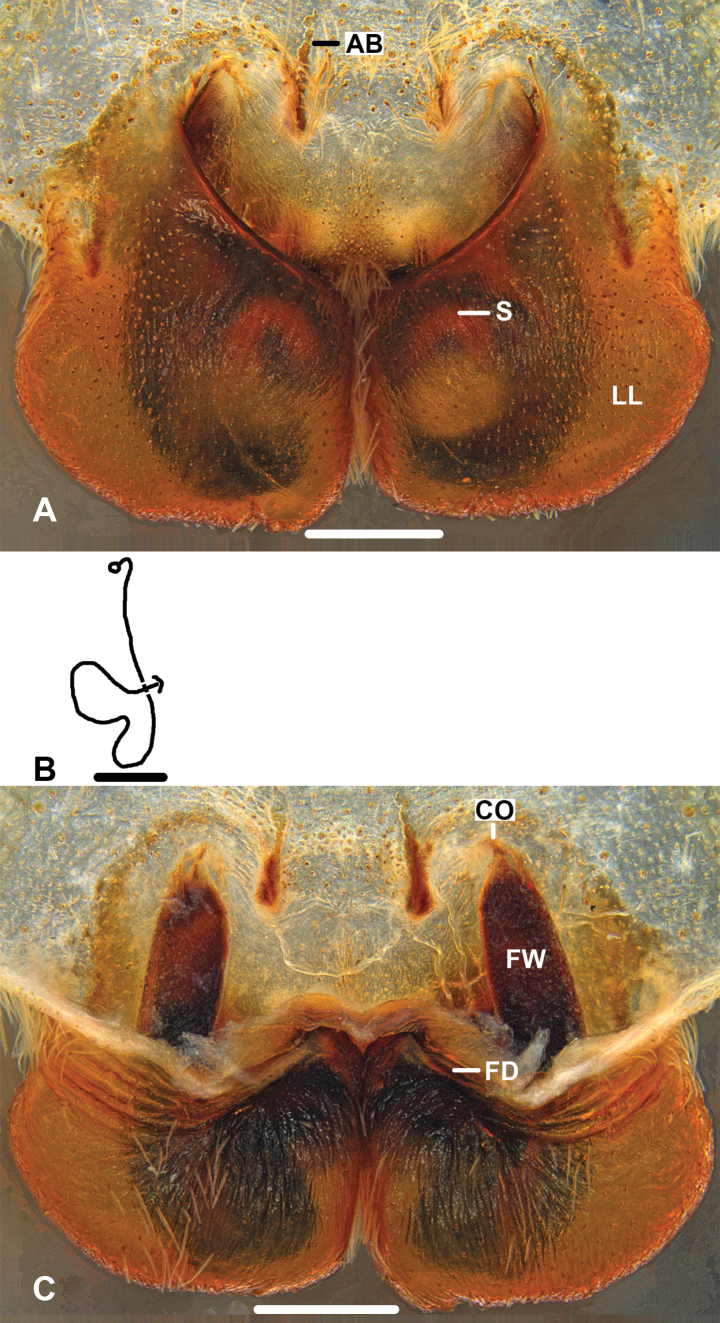
*Pseudopodafengtongzhaiensis* Jäger & Liu, sp. nov., holotype, female **A** epigyne, ventral **B** schematic course of IDS, dorsal **C** vulva, dorsal. Scale bars: 0.5 mm.

***Epigyne*** (Fig. 3A–C): As in diagnosis. EF wider than long, with distinct AB. Anterior margins of LL distinctly curved and forming a broad V-shaped, and resembling a heart, in ventral view. FW of IDS well developed, covering entire S, large parts of FW covered by LL in dorsal view. FD long and narrow, suited medially.

**Figure 4.**
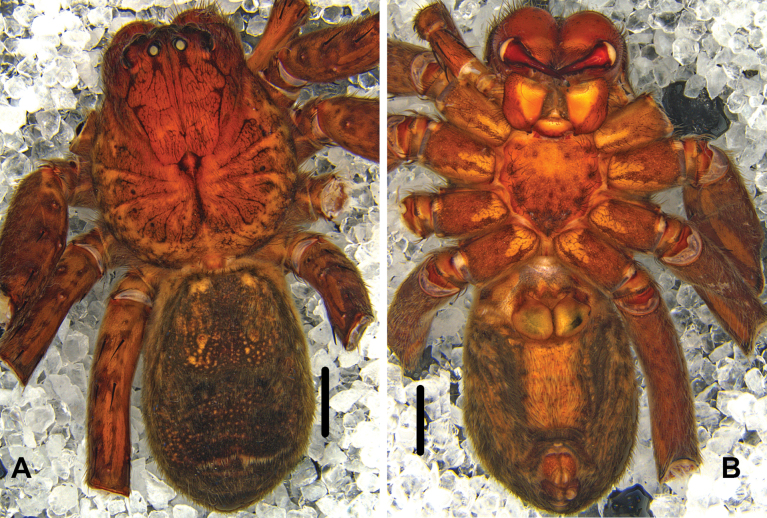
*Pseudopodafengtongzhaiensis* Jäger & Liu, sp. nov., female habitus (**A** dorsal **B** ventral). Scale bars: 2 mm.

***Colouration*** (Fig. 4A, B): DS reddish-brown with dark spots, margin with distinct marks. Fovea and striae distinctly marked and with a few dark dots. OS dorsally dark brown, with lots of yellow and small dots, and several big dots in anterior part, with a transversal yellow line in posterior part. OS ventrally brown, margins with dark hairs, with a yellow region in middle part.

**Male**: Unknown.

##### Variation.

Females (*N* = 3): body length 15.1–16.3, DS length 7.1–8.0, OS length 8.0–8.3.

##### Remarks.

This species may potentially be conspecific with *P.acutiformis* Zhang, Jäger & Liu, 2023 (Zhang et al. 2023), as they share the same habitat. However, there are notable differences in the coloration and patterns on the dorsal side of the females compared to the males of *P.acutiformis*, suggesting they might be distinct species. Additionally, another species, *P.flexa* Zhang, Jäger & Liu, 2023 (Zhang et al. 2023), is also located in this Reserve. Resolution of these ambiguities will depend on future research and findings.

##### Distribution.

China (Sichuan Province) (Fig. 9).

#### ﻿ Pseudopoda inthanonensis

Taxon classificationAnimaliaAraneaeSparassidae

Jäger & Liu sp. nov.

107E7325-E5B5-528B-89D1-5343D7B61943

https://zoobank.org/E105C872-E060-4202-AA79-6C5DB6EAC7E4

Figs 5 , 6 , 9

##### Type material.

***Holotype*** female: Thailand, Chiang Mai Province: Doi Inthanon National Park, [18°35′24″N, 98°28′48″E], 1680 m, 24 October 2002, P. Dankittipakul leg. (MHNG, THI0068).

##### Etymology.

The specific name is derived from the type locality, the Doi Inthanon National Park; adjective.

##### Diagnosis.

The female of *P.inthanonensis* Jäger & Liu, sp. nov. can be distinguished from those of all other *Pseudopoda* species by the posterior margins of LL with a distinct inverted V-shaped indentation at the middle part.

**Female** (THI0068): ***Measurements***: Medium sized. Body length 12.7, DS length 4.9, width 4.2, OS length 7.8, width 4.5. Eyes: AME 0.24, ALE 0.34, PME 0.27, PLE 0.32, AME–AME 0.19, AME–ALE 0.09, PME–PME 0.21, PME–PLE 0.38, AME–PME 0.30, ALE–PLE 0.26, CHAME 0.36, CHALE 0.27. ***Spination***: Pp 131, 001, 2121, 1004; Fe I–II 323, III 322, IV 331; Pa I–IV 101; Ti I–IV 2026; Mt I–II 2024, III 3024, IV 3036. ***Measurement of palps and legs***: Pp 7.8 (2.3, 1.8, 1.0, –, 2.7); I 23.8 (7.0, 2.3, 6.9, 6.1, 1.5), II 28.4 (8.1, 2.5, 8.4, 7.3, 2.1), III 18.4 (6.0, 1.5, 5.1, 4.7, 1.1), IV 24.3 (7.3, 1.8, 6.3, 6.9, 2.0). Leg formula: II-IV-I-III. Promargin of chelicerae as in *P.baimai* Jäger & Liu, sp. nov., cheliceral furrow with c. 27 denticles.

**Figure 5.**
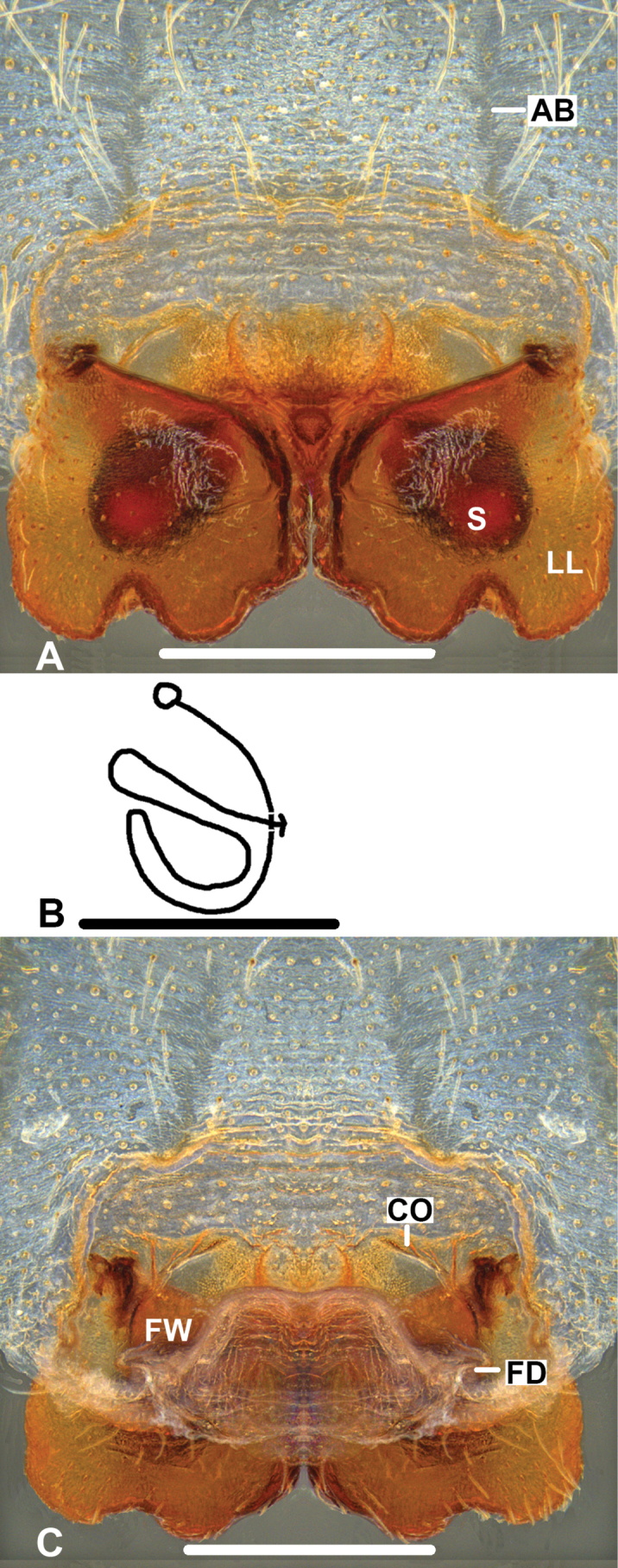
*Pseudopodainthanonensis* Jäger & Liu, sp. nov., holotype, female **A** epigyne, ventral **B** schematic course of IDS, dorsal **C** vulva, dorsal. Scale bars: 0.5 mm.

***Epigyne*** (Fig. 5A–C): As in diagnosis. EF wider than long, AB long, but indistinct. LL narrow, with anterior margins slightly curved. S oval-shaped in ventral view, covered by FW and posterior part of LL in dorsal view. FD long, suited medially.

**Figure 6.**
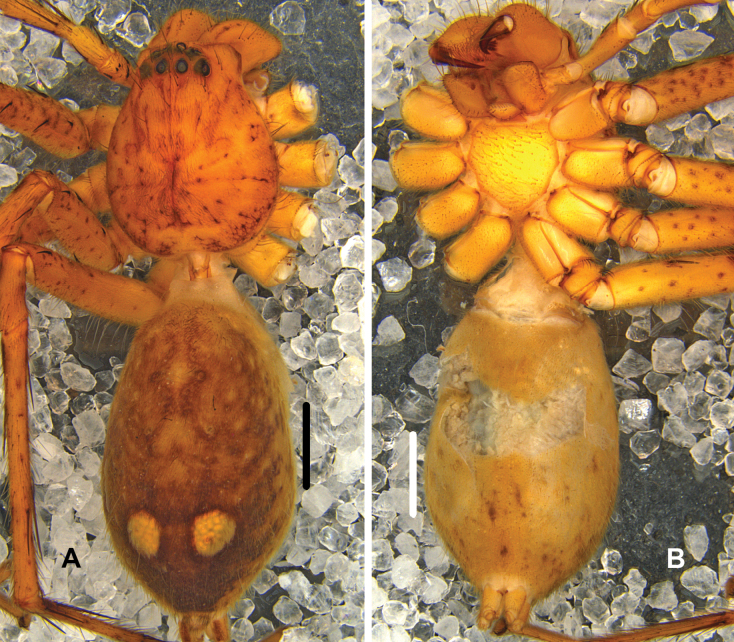
*Pseudopodainthanonensis* Jäger & Liu, sp. nov., female habitus (**A** dorsal **B** ventral). Scale bars: 2 mm.

***Colouration*** (Fig. 6A, B): DS reddish-brown with dark spots. Margins with dash dark dots. Fovea and striae distinctly marked. OS dorsally brown, with several dots at anterior part, with two round patches at the posterior half. OS ventrally yellow, with several brown and irregular marks.

**Male**: Unknown.

##### Remarks.

It is possible that this species is conspecific with *P.columnacea* Zhang, Jäger & Liu, 2023 (Zhang et al. 2023) due to their shared habitat. However, significant variations in the color and patterns on the dorsal side of the females compared to the males of *P.columnacea* suggest they could be different species. Clarifying these uncertainties will require additional research and future discoveries.

##### Distribution.

Thailand (Chiang Mai Province) (Fig. 9).

#### ﻿ Pseudopoda kavanaughi

Taxon classificationAnimaliaAraneaeSparassidae

Zhang, Jäger & Liu, 2023

9434B03A-D3F0-5F9F-83CC-4CEEBDF6199F

Figs 7 , 8 , 9

Pseudopoda kavanaughi Zhang, Jäger & Liu, 2023: 149, figs 135A–C, 136A, B (Holotype male from Yunnan Province, China, deposited in CAS, examined).

##### Material examined.

China, Yunnan Province: 1 female, Gongshan County, Dulongjiang Township, 2.3–3.3 km south of Longyuan Village, 28°0′19″N, 98°19′42″E, 1690 m, 2 November 2004, D.H. Kavanaugh leg. (CAS, CAS0035); 1 female, Gongshan County, Dulongjiang Township, Dizheng Wang, across Dulongjiang from Dizhengdang, 28°5′12″N, 98°19′42″E, 1910 m, 20 October 2004, D.H. Kavanaugh leg. (CAS, CAS0034).

##### Diagnosis.

The female of this species is similar to *P.tianpingensis* Zhang, Jäger & Liu, 2023 (Zhang et al. 2023) by: 1) anterior margins of LL U-shaped and almost parallel to posterior margins; and 2) sclerotised part of IDS oval-shaped in ventral view. It can be recognised by the sclerotised part of S forming “八” -shape, extending in oblique downward axis in ventral view (almost parallel to anterior margins and posterior margins, extending in oblique upward axis in ventral view in *P.tianpingensis*).

**Female** (CAS0034): ***Measurements***: Small sized. Body length 8.2, DS length 3.8, width 3.5, OS length 4.4, width 3.3. Eyes: AME 0.22, ALE 0.35, PME 0.25, PLE 0.33, AME–AME 0.14, AME–ALE 0.08, PME–PME 0.18, PME–PLE 0.38, AME–PME 0.29, ALE–PLE 0.24, CHAME 0.36, CHALE 0.27. ***Spination***: Pp 131, 101, 2121, 1014; Fe I–III 323, IV 321; Pa I–IV 101; Ti I–III 2224, IV 2226; Mt I–II 1014, III 2024, IV 3036. ***Measurement of palps and legs***: Pp 5.0 (1.5, 1.0, 0.7, –, 1.8); I 12.6 (3.5, 1.5, 3.7, 2.9, 1.0), II 15.4 (4.3, 1.8, 4.5, 3.3, 1.5), III 10.2 (3.1, 0.9, 2.8, 2.6, 0.8), IV 13.1 (3.9, 1.2, 3.4, 3.1, 1.5). Leg formula: II-IV-I-III. Promargin of chelicerae as in *P.baimai* Jäger & Liu, sp. nov., cheliceral furrow with c. 16 denticles.

**Figure 7.**
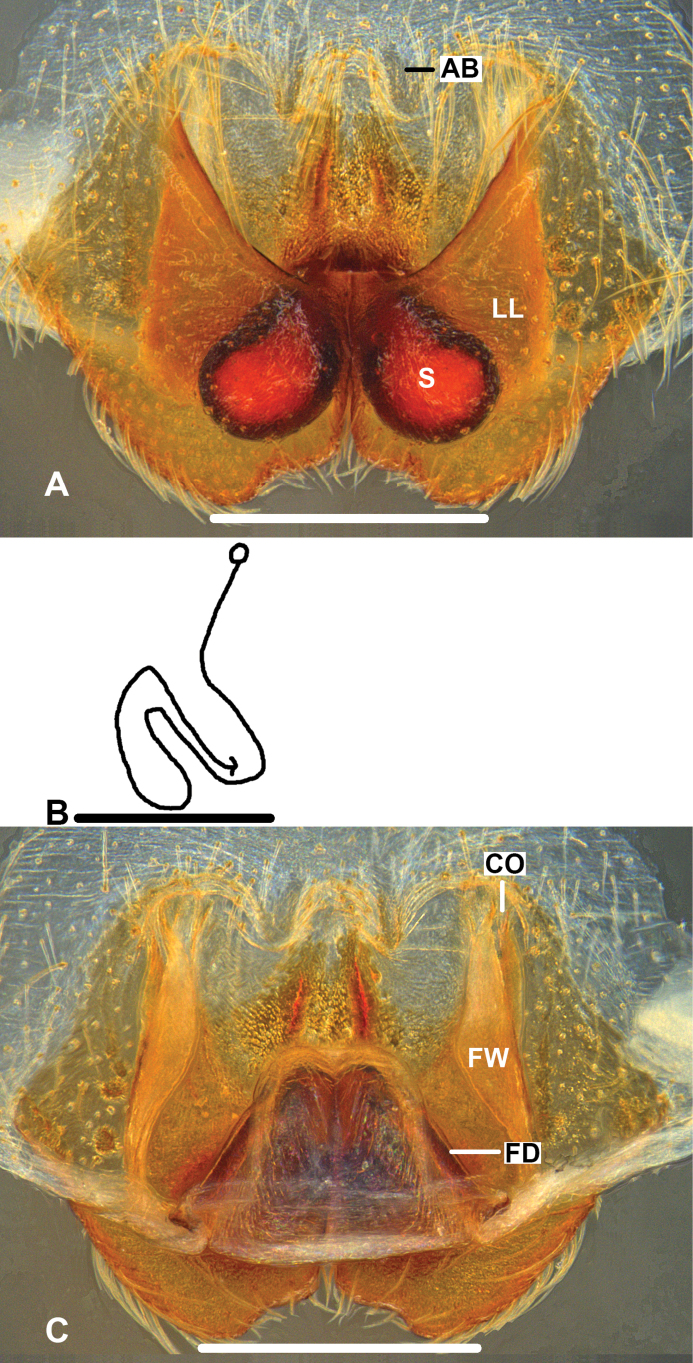
*Pseudopodakavanaughi* Zhang, Jäger & Liu, 2023, female **A** epigyne, ventral **B** schematic course of IDS, dorsal **C** vulva, dorsal. Scale bars: 0.5 mm.

***Epigyne*** (Fig. 7A–C): As in diagnosis. EF wider than long, with short AB. S clearly visible, and slightly bent to anterior margins to LL in ventral view, all covered by FW and posterior part of LL in dorsal view. FD long, suited medially.

**Figure 8.**
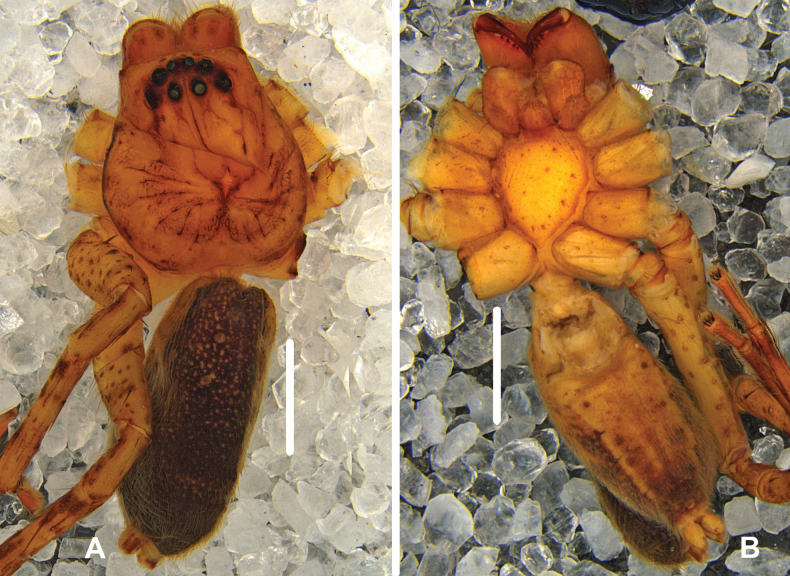
*Pseudopodakavanaughi* Zhang, Jäger & Liu, 2023, female habitus (**A** dorsal **B** ventral). Scale bars: 2 mm.

***Colouration*** (Fig. 8A, B): DS reddish-brown with dark dots. Fovea and striae distinctly marked. OS dorsally reddish-brown with lots of black dots, ventrally brown with several black marks, with several reddish-brown patches at posterior part.

**Male**: For details see Zhang et al. (2023).

##### Variation.

Female (*N* = 1): body length 7.7, DS length 3.2, OS length 4.5.

##### Remarks.

This female’s location is close to that of the male of *P.kavanaughi*. Although there are slight differences in colouration, we consider it the conspecific female.

##### Distribution.

**Figure 9.**
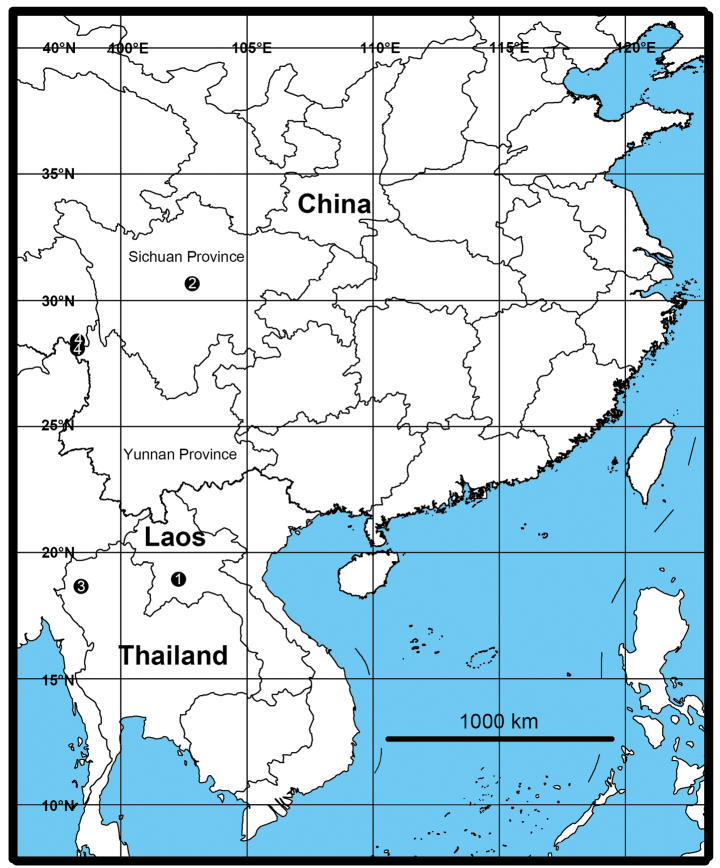
Distribution map of the four species from the genus *Pseudopoda*. The numbers represent the different species. **1***P.baimai* Jäger & Liu, sp. nov. **2***P.fengtongzhaiensis* Jäger & Liu, sp. nov. **3***P.inthanonensis* Jäger & Liu, sp. nov. **4***P.kavanaughi* Zhang, Jäger & Liu, 2023.

China (Yunnan Province) (Fig. 9).

## Supplementary Material

XML Treatment for Pseudopoda

XML Treatment for Pseudopoda baimai

XML Treatment for Pseudopoda fengtongzhaiensis

XML Treatment for Pseudopoda inthanonensis

XML Treatment for Pseudopoda kavanaughi

## ﻿Additional information

### Data availability

All of the data that support the findings of this study are available in the main text.
